# Experiences and perceptions of nurses participating in an interprofessional, videoconference-based educational programme on concurrent mental health and substance use disorders: a qualitative study

**DOI:** 10.1186/s12912-022-00943-w

**Published:** 2022-07-04

**Authors:** Gabrielle Chicoine, José Côté, Jacinthe Pepin, Louise Boyer, Geneviève Rouleau, Didier Jutras-Aswad

**Affiliations:** 1 Faculty of Nursing, Université de Montréal, Montreal, Quebec (QC) Canada; 2 Université de Montréal Hospital Research Centre, Montreal, QC Canada; 3 Research Chair on Innovative Nursing Practices, Faculty of Nursing, Université de Montréal, Montreal, QC Canada; 4 Équipe FUTUR-FRQSC, Faculty of Nursing, Université de Montréal, Montreal, QC Canada; 5 Centre for Innovation in Nursing Education, Faculty of Nursing, Université de Montréal, Montreal, QC Canada; 6 Women’s College Hospital, Toronto, Ontario (ON) Canada; 7 Faculty of Medicine, Department of Psychiatry and Addiction, Université de Montréal, Montreal, QC Canada; 8 Centre of Excellence and Collaboration in Co-Occurring Disorders, Université de Montréal Hospital Center, Montreal, QC Canada

**Keywords:** Competencies, Competency development, Continuing education, Co-occurring disorders, Interpretive description, Project ECHO, Virtual communities

## Abstract

**Background:**

Individuals with co-occurring mental health and substance use disorders (i.e., concurrent disorders) have complex healthcare needs, which can be challenging for nurses to manage. Providing optimal care for this subpopulation requires nurses to develop high-level competencies despite limited resources at their disposal and the isolated settings in which many of them work. The Extension for Healthcare Community Outcomes (ECHO®) is a promising collaborative learning and capacity building model that uses videoconference technology to support and train healthcare professionals in the management of complex and chronic health conditions. The aim of this study was to explore the experiences and perceptions of nurses participating in a Canadian ECHO programme on concurrent disorders about the competencies they developed and used in their clinical practice, and which factors have influenced this process.

**Methods:**

The study was qualitative, guided by an interpretive description approach. Individual semi-structured interviews were held with ten nurses who had participated in the programme between 2018 and 2020. A thematic analysis was conducted iteratively using an inductive approach to progressive data coding and organization.

**Results:**

Four themes and eighteen sub-themes were identified. During their participation in ECHO, the nurses perceived as having further developed eight clinical nursing competencies. Nurses viewed ECHO as a unique opportunity to open themselves to their peers’ experiences and reflect on their own knowledge. Learning from experts in the field of concurrent disorders helped them to build their confidence in managing complex clinical situations. The nurses’ sense of belonging to a community further enhanced their engagement in the programme, and learning was facilitated through the programme’s interprofessional environment. Nevertheless, the lack of contextualized educative content linked to local realities, the limited resources in concurrent disorders, and time constraints were experienced as factors limiting competency development.

**Conclusions:**

ECHO is a promising alternative to conventional, in-person continuing education programmes to improve the development of advanced competencies among nurses providing care to individuals with chronic and complex health conditions. These findings can inform clinicians, educators, researchers, and decision makers who are developing, implementing, evaluating, and escalating future educational interventions in the field of CDs.

**Supplementary Information:**

The online version contains supplementary material available at 10.1186/s12912-022-00943-w.

## Introduction

Concurrent disorders (CDs) refer to the simultaneous occurrence of mental health and substance use disorders. Individuals with CDs often experience poorer physical health and social outcomes, and greater psychological distress than do people with a single disorder [1]. Besides their high prevalence, CDs are strongly associated with an increased risk of suicide, poorer compliance to treatment, violence/delinquency, as well as greater risks for infection such as HIV and hepatitis [2]. Further, people with CDs have poorer social outcomes including homelessness, social isolation, stigma and care access inequities [3]. Failure to address the complex healthcare needs of this subpopulation can lead to high relapse rates, long hospital stays and increased health care costs [4, 5].

In Canada, most people experiencing CDs are managed within primary healthcare settings [1], yet the majority of nurses working in these clinical contexts have limited or no specific education to do so [6]. Previous studies highlighted that nurses often perceived themselves as deskilled and ill-equipped, particularly for competencies such as screening mental disorders in active users, managing recurrent psychotic symptoms, offering appropriate interventions in crisis situation and coordinating care between clinical team and agencies [7]. Research has also indicated that some nurses hold counterproductive and stigmatizing attitudes when caring for individuals with problematic alcohol or drug use [8]. Moreover, feelings of frustration, hopelessness, exhaustion, powerlessness, and isolation are common among nurses who encounter people living with CDs [9, 10].

These challenges contribute to the pressing need for further educational opportunities that align with public health organizations’ guidelines on integrated mental health and substance use care in order to help nurses develop their competencies in this field [11]. To this end, this paper reports on an innovative videoconferencing educational programme based on the Extension for Community Healthcare Outcomes (ECHO®) model that seeks to improve the knowledge and competencies of nurses in safely and effectively managing individuals with CDs.

## Background

Continuing professional education is universally agreed upon by researchers, clinical leaders, and practitioners as a fundamental strategy for enhancing and sustaining the competencies of nurses [12]. For instance, professional education opportunities all through their career continuum have been identified as an important means of fostering life-long learning and practice renewal [13]. In recent decades, advances in technology have brought new ways of offering a wide range of continuing educational interventions. Technology-enabled educational interventions have become an increasingly popular alternative because of their ease of delivery, low cost, high accessibility, and greater flexibility [14]. One such promising educational intervention, the ECHO model, uses videoconference technology to offer ongoing support and education to healthcare professionals managing patients with complex and chronic health conditions through scheduled case-based discussions held remotely. The model was launched in 2003, in the United States, under the name of Project ECHO® at the University of New Mexico, in Albuquerque, New Mexico; its initial objective was to improve access to Hepatitis C treatment in rural New Mexico [15]. Since then, there have been over 590 replications of ECHO in 34 countries, covering up to 68 disease-related topics [16]. ECHO typically involves pairing an interprofessional team of experts from academic healthcare centres with many other healthcare professionals, regardless of geographical barriers. It provides them with the opportunity to learn from one another, create a knowledge network, and build a stronger team dynamic.

There is growing evidence showing the ECHO model’s acceptability, feasibility, and positive impact on both healthcare professionals and patients’ outcomes [17, 18]. With respect to provider-related outcomes, one recent systematic review highlighted that most of the existing empirical research on ECHO have shown favourable results across three domains: satisfaction, increased knowledge, and increased confidence [19]. Arguably, the most desirable outcomes are the changes in clinical practice, by virtue of the capacity-building orientation of the ECHO model. Five studies have examined the impact of ECHO on behaviour change among healthcare professionals, based on data gathered via medical chart reviews or self-reported surveys [20–24]. Overall, those studies suggested that healthcare professionals had or would have altered care provision as a result of presenting patient cases and being offered concrete recommendations from both peers and experts. For example, Komaromy et al. [22] found that 77% of participants from an ECHO programme on behavioural health reported that case-based discussion changed their patient care plan. Likewise, Catic and colleagues [20] observed from an ECHO programme in geriatric long-term care that recommendations for treatment were incorporated by presenters 89% of the time.

Despite this breadth of evidence, the nursing perspective is sparse and under-represented within the ECHO literature, even though nurses play a critical role in the early recognition and management of CDs because they have the most frequent contact with CD patients and they are centrally responsible for ensuring the continuity of care [25]. Indeed, only two studies of ECHO programmes focusing on nurses’ outcomes and/or perceptions have been published to date [26, 27]. In the first study, a formative evaluation of an ECHO programme was conducted among 34 primary care nurses and reported that most respondents agreed that the educational content was very or extremely meaningful to their work [26]. In the second study, a six-month prospective longitudinal cohort study (*n* = 28) was piloted in a community palliative care setting and found that nurses’ mean score for knowledge and skills improved significantly—by 11.3% from baseline to post-ECHO [27]. This study also showed significant improvements in all domains of nurses’ self-efficacy, with the greatest degree of change in the domain of “symptom management, maintaining comfort and wellbeing”.

While these results lend support to the benefits of ECHO, there remain important gaps in our understanding of how ECHO contributes to nursing competency development, including a dearth of studies in the field of CDs [22, 28–32]. In this regard, it has been robustly documented that little scientific attention has focused on what factors influence competency development among ECHO participants [16], while such factors are crucial to leveraging potential strategies that might reinforce learning and sustain changes in clinical practice. Therefore, we aimed at studying the competency development of nurses during their participation in a Canadian ECHO programme for CD management (ECHO-CD) and at exploring the factors that have influenced this process, whether positively or negatively.

### Philosophical and conceptual underpinnings: a socio-constructivist approach to competency development

A holistic, context-bound, and experientially based conceptual perspective of competency development informed this study [33]. Situated within a socio-constructivist epistemology, competency development is understood as an evolutive and infinite process of learning occurring within the context of social and environmental interactions [33]. This perspective also suggests that learning is a subjective experience, whereby nurses are actively engaged in building up new knowledge upon existing knowledge and personal experiences to develop themselves in a unique way [34]. A competency is viewed as “a complex knowledge in action” [34], based on the effective mobilization and combination of a variety of coordinated resources (e.g., skills, attitudes, material resources), each of which is fundamental for competent nursing practice in specific situations. This definition emphasizes that competencies are inherently context-bound and, as such, allow nurses to constantly adapt their practice, both to the unique needs of their patients and as they encounter new clinical situations. Hence, learning and competency development are intertwined; nurses progressively develop their competencies as they engage in meaningful learning situations throughout their professional career.

## Methods

This qualitative study was part of a larger mixed methods research project that sought to develop a comprehensive understanding of the impact of a Canadian ECHO programme for CD management on the competency development and clinical practice of nurses [35]. It was guided by the following two-fold research question:

- How did the nurses implement, in their clinical practice, the competencies they perceived as having developed through their participation in ECHO-CD, and what factors have influenced this process?

This research article was developed and organized in accordance with the Consolidated Criteria for Reporting Qualitative Research (COREQ) checklist [36] (Additional file 1).

### Research design

This study employed an interpretive description (ID) methodology [37]. ID offers an appropriate and theoretically flexible approach to collecting, analysing, and interpreting qualitative data within applied health disciplines by addressing complex experiential questions while producing practical evidence [38]. Within this study, ID was used to structure the research procedures, illuminate themes and patterns/discrepancies in the perspectives of the participants, and provide an integrative description of the learning experience obtained by the nurses through their participation in ECHO-CD. Consistent with the socio-constructivist perspective that was selected to guide this study, the research team played a role in recognizing and constructing interpretations from participants’ multiple perspectives.

### Setting and educational intervention

The ECHO programme for CD management was launched in September 2018 at a quaternary academic hospital centre in the province of Quebec, Canada. It was developed by a multidisciplinary team of researchers and healthcare professionals (i.e., psychiatrists, nurses, occupational therapists, social workers) with expertise in the field of CDs. Table 1 describes ECHO-CD using the Guidelines for Reporting Evidence-based practice Educational interventions and Teaching (GREET) [44]. More details regarding the educational intervention can be found in the study protocol, which has been published elsewhere [35], or directly on the programme’s website (https://ruisss.umontreal.ca/cectc/services/echo-troubles-concomitants/). This study collected data from the nurses who participated in one or more of the first 2 cycles of the programme (curricula for 2018–2019 and 2019–2020), thus during the programme’s implementation and expansion.

**Table 1:** Description of the educational intervention using the GREET

BRIEF NAME: ECHO programme for CD management (ECHO-CD)		
**1**	**INTERVENTION**	
**WHY - this educational process**		
**2**	**THEORY**	This educational intervention was developed according to the ECHO model [39, 40], which is rooted in three established social, learning theories: 1) Bandura’s Social Cognitive Theory [41]; 2) Lave and Wenger’s Situated Learning Theory [42]; and 3) Wenger’s Theory of Communities of Practice [43].
**3**	**LEARNING OBJECTIVES**	The educational intervention embraced three distinct learning objectives: 1) to enhance participants’ knowledge in CD EBP; 2) to amplify participants’ competencies in addressing CDs and facing complexity; and 3) to build a learning community in which healthcare professionals can receive support in working with challenging situations of patients with CDs. The educational intervention also included specific learning objectives based on the case-based discussion and the didactic presentations for each session of a given curriculum. These learning objectives were developed to match the NICE 2016 guidelines on CD care [11]. Specific learning objectives were applicable to all professional groups. An example of the specific learning objectives for the 2018–2019 curriculum can be found in the study protocol published elsewhere [35].
**4**	**EBP CONTENT**	Each session included a didactic presentation that consisted of a specific CD EBP topic. The topics covered mental health and psychiatric issues (e.g., psychotic disorders, anxiety disorders, eating disorders), addiction care and treatment (e.g., opioid use disorder, withdrawal management), co-occurring medical issues (e.g., HCV), as well as other psychosocial topics (e.g., homelessness, legal and ethical issues, referral pathways). It also included broader CD-related topics, such as basics in integrated care treatment, core values, attitude, and relational skills, and planning and coordinating care between healthcare professionals, teams, and agencies.
**WHAT**		
**5**	**MATERIALS**	**Materials provided to learners:** - At the time of their registration, participants were provided with an electronic document explaining the educational intervention’s purpose, the sessions’ structure and functioning, and the learning objectives and activities. - One week prior to each session, an electronic document detailing the clinical situation to be discussed was emailed to all participants. This document has predetermined sections, which were filled out by the healthcare professionals presenting the clinical situation (i.e., patient case). - Didactic presentations were supported with a PowerPoint presentation and shared with participants via email after each session. - The programme has its own website, which offers several CD resources that participants can consult at any time. - A scientific librarian emailed scientific articles and clinical tools related to CDs each month. - A written summary combining recommendations and guidance from the team of experts was sent to healthcare professionals (or team of healthcare professionals) who had presented a clinical situation. This electronic document generally consisted of interventions to add to their patients’ care plan. **Materials used for instructors:** A paper-version document that details the ECHO model learning principles (i.e., learning methods and educational strategies) was given to each member of the team of experts as guidance on EBP teaching methods. This document also included a step-by-step approach on how to replicate the ECHO model in other contexts. **VC equipment:** To run a session online, the team of experts used a *Logtech Group ConferenceCam* kit that is connected to a Lenovo Windows PC with two 55″ screen mounted on a support. In this study, nurses were able to join videoconference sessions via a desktop or laptop computer, phone, tablet or any other mobile device. Nurses were equipped by their employer for the minimum technical equipment required to run a videoconference session online (i.e., desktop or laptop computer, internet connection, speakers, and microphones). However, some nurses did not have access to a webcam or HD cam.
**6**	**EDUCATIONAL STRATEGIES**	During each session, the following three educational strategies were used, concurrently: - **Case-based discussion:** For each session, a clinical situation was chosen by a healthcare professional (or team of healthcare professionals), and then presented to all participants. Prior to the session, the healthcare professional was asked to prepare a summary of the clinical situation by detailing the patient’s biopsychosocial needs, and by identifying questions for the group to consider about that clinical situation. Following that, a discussion period allowed for questions, reflections, and sharing of knowledge and personal experiences. Lastly, recommendations and guidance from the team of experts and participants were provided verbally during the session and then in a written summary to the healthcare professional (or team of healthcare professionals) who presented the clinical situation. - **Traditional lecture:** Didactic presentations about CD EBP. - **Reflective practice:** In the weeks to months after the case-based discussion, some participants were asked to present the chosen clinical situation again. During this follow-up discussion, the implementation and the impact of the recommendations provided during the previous session were reviewed and assessed. Participants also had the opportunity to complete an online test of their CD CDs every six months. This provided them with feedback from the team of experts on their learning needs. In this qualitative study, research interviews with nurses allowed them to reflect on their learning progress during their ECHO-CD participation, and how it contributed to their clinical practice.
**7**	**INCENTIVES**	Continuing education credits were given to participants after each completed session.
**WHO PROVIDED**		
**8**	**INSTRUCTORS**	**Instructors:** The team of experts included psychiatrists, physicians with a specialization in substance-use disorders, registered nurses and a clinical nurse specialist, pharmacists, social workers, psychologists, occupational therapists, and a scientific librarian. **Other professionals included in the intervention:** - In case of a specific medical issue, specialists from the quaternary hospital centre were invited to join the team of experts for further guidance (e.g., hepatologist, physician with expertise in HIV treatment). - Each session, a project manager assisted the team of experts to mediate the participants’ interactions. This involved answering the participants’ questions in the forum’s app and ensuring that each participant had the opportunity to ask questions or share their knowledge, experience, and/or ideas. - Additionally, a computer scientist offered in-person support during each session to resolve any technical issues that could arise during the session. **Experience and expertise:** All healthcare professionals from the team of experts had expertise in CDs or at least six months of experience in working with CD patients. Registered nurses from the expert team had at least a bachelor’s degree and at least six months of clinical experience in CD care. According to their discipline, the healthcare professionals on the expert team had different specializations such as motivational interviewing, relapse prevention, cognitive and behavioural therapy, working with vulnerable populations (e.g., youth, homeless people, pregnant women), Hepatitis C treatment and treatment for opioids use disorders. **Roles:** - Facilitator: During each session, the same psychiatrist on the expert team acted as a facilitator. This role consisted of introducing each member of the expert team, making sure that all participants had time to introduce themselves, summarizing expert and peer recommendations at the end of a session, and ensuring that the session went smoothly and that the schedule was followed. The facilitator also provided feedback to participants throughout the sessions. - Team of experts: Healthcare professionals from the team of experts are invited to ask questions regarding the clinical situation for further information and/or clarifications. They also provide recommendations and/or feedback during the course of a given session, according to their own discipline and area of expertise. At each session, a healthcare professional from the team of experts delivered a didactic presentation on CD EBP. **Training related to the educational intervention provided to instructors:** As part of a requirement for ECHO-affiliated programmes, two healthcare professionals from the team of experts attended a four-day immersion training by the ECHO Ontario Mental Health (ECHO-OMH) programme at the Centre for Addictions and Mental Health (CAMH) in the province of Toronto, Canada [32]. The goal of the immersion training was to offer guidance on how to replicate the ECHO model in other contexts and to ensure that replicated ECHO programmes are delivered according to the highest standards of continuing education. Training on learning methods, teaching strategies, and core pedagogical skills was provided during this immersion. The immersion training session was originally developed at the ECHO Institute in the state of New Mexico, US, to ensure fidelity between the ECHO model and future replications.
**HOW**		
**9**	**DELIVERY**	**Modes of delivery:** The educational intervention was exclusively provided online through simultaneous videoconference sessions. Learning activities were held as a group. **Ratio:** There were no formal limits on the number of online participants for each session. Each curriculum had up to 200 registrants, with an average of 50 to 60 participants connected at any one time, and a minimum of four experts with different interdisciplinary backgrounds to ensure that recommendations were tailored to a wide range of professional groups. **Sequence of learning activities:** Each curriculum included an orientation session to familiarize participants with the educational intervention’s structure and learning objectives, and the videoconference technology. Then, each subsequent session had five main learning activities, which took place into the following sequence: 1) a 10-minute introduction, in which the team of experts and the participants introduced themselves; 2) a presentation about a clinical situation (15 minutes); 3) a discussion period regarding the clinical situation (30 minutes); 4) a period for clinical guidance and recommendations (15 minutes); and 5) a didactic presentation, including a lecture and questions (20 minutes).
**WHERE**		
**10**	**ENVIRONMENT**	**Location:** The team of experts (the “Hub”) delivered the educational intervention from a conference room in the quaternary academic hospital centre, located in the province of Quebec, Canada. Participating health care professionals (the “Spokes”) were located in different urban and rural areas across the province and joined the sessions from their workplace or home. **Technical environment:** The Zoom platform.
**WHEN AND HOW MUCH**		
**11**	**SCHEDULE**	**Number of sessions:** Each curriculum included 20 sessions from September to June. Participants had the opportunity to register for more than one cycle. **Frequency:** Every two weeks. **Timing and duration:** 90 minutes, from 12:00 p.m. to 1:30 p.m.
**12**	**FACE-TO-FACE CONTACT WITH INSTRUCTORS AND/OR SELF-DIRECTED LEARNING ACTIVITIES**	Each session consisted of virtual face-to-face contact between the team of experts and other participants. Self-directed activities consisted of clinical guidance, tailored recommendations, and feedback.
**PLANNED CHANGES**		
**13**	**SPECIFIC ADAPTATION FOR THE LEARNERS**	The content of the educational intervention was adapted to the participants’ needs as follows: - In order to adapt content to participants’ requests and learning needs, no specific topics were planned for the last two didactic presentations of each curriculum. The topics of these two didactic presentations were chosen based on the participants’ responses in the after-session feedback questionnaires. - If a specific health issue generated questions, a scientific librarian provided participants with further resources and/or information during or after the session.
**UNPLANNED CHANGES**		
**14**	**MODIFICATIONS MADE TO THE EDUCATIONAL INTERVENTION DURING THE COURSE OF THE STUDY**	During the COVID-19 pandemic, the following modifications were made to the 2019–2020 curriculum: - Content: Two didactic presentations on COVID-19 were developed and later presented to participants (i.e., management of CDs and COVID-19 during hospitalization, and issues related to people with substance use disorders and COVID-19). - Environment: Healthcare professionals from the team of experts attended the sessions in separate rooms instead of being grouped in a larger conference room.
**HOW WELL**		
**15**	**ATTENDANCE**	Participation in each session was not mandatory. However, the frequency of nurses’ session attendance was tracked as part of a larger mixed methods research project [35].
**16**	**PROCESSESS TO DETERMINE WETHER THE MATERIALS (item 5) AND EDUCATIONAL STRATEGIES (item 6) WERE DELIVERED AS PLANNED**	In this qualitative study, nurses were invited to describe their experiences and perceptions regarding the educational intervention and to reflect on their own learning during individual semi-structured interviews. Also, an ECHO-CD Committee was implemented at the quaternary academic health centre for continuing programme improvement. Within this committee, healthcare professionals who participated in the first two cycles of ECHO-CD were invited to provide feedback and suggestions. These were later used to adapt the programme to their learning needs and local realities.
**17**	**EXTENT TO WHICH THE EDUCATIONAL INTERVENTION WAS DELIVERED AS SCHEDULE**	Both curricula (i.e., 2018–2019 and 2019–2020) were delivered as scheduled.

*CDs* Concurrent disorders, *EBP* Evidence-based practice, *ECHO-CD* Extension for Community Healthcare Outcomes programme for concurrent disorder management, *HCV* Hepatitis C Virus, *HD* High definition, *HIV* Human Immunodeficiency Virus, *NICE* National Institute for Health and Care Excellence, *US* United States, *VC* Videoconference

### Participants and recruitment

The potential study population consisted of 65 nurses who registered for ECHO-CD during its first and second cycle. Nurses were initially informed of the study by email at the time of their programme registration and by a research coordinator during the virtual sessions. The inclusion criteria consisted of nurse participants in the programme’s 2018–2019 and 2019–2020 curricula (*N = 65*) who attended at least one virtual session and consented to the research. Nurses playing all types of professional roles were eligible to participate in the study: registered nurses, nurse practitioners, assistant head nurses, clinical nurse specialists, auxiliary nurses, etc. Each potential participant was recruited by the primary author (GC; female registered nurse and PhD candidate in the field of nursing science with previous experience in qualitative research and conducting interviews) by email or by phone, according to the contact preference indicated in each nurse’s programme registration. A total of 32 nurses met the inclusion criteria and were invited to participate. The recruitment took place between May 2020 and July 2020.

In accordance with the ID approach—which recognizes that subjective human experience can theoretically possess infinite variation—data saturation was not a desired outcome of this study. Rather, we focused on obtaining a deeper understanding of participants’ experiences, and on ensuring that the data we gathered was rich enough to answer our research question. More specifically, sampling decisions were made during the process of data collection, based on obtaining variation between the nurses’ characteristics (i.e., year of participation in the programme, session attendance, academic and professional background, work setting) or when no new information emerged from the participants. In this study this occurred after the inclusion of 10 participants.

Table 2 provides an overview of the demographics and practice profile of the 10 nurse participants. The sample was comprised mainly of women (*n* = 9), with a mean age of 39.4 years (*SD* = 3.3). All the nurses held more than 5 years of clinical experience, and most were case managers (*n =* 7).

**Table 2:** Demographics and practice profile of the study sample (*n* = 10)

Characteristics	Total (***n***) or mean (***SD***)
**Gender**	
Female	9
Male	1
**Mean age**	39.4 (3.3)^a^
**Degree earned**	
Bachelor’s degree	4
Master’s degree	6
**Professional role**	
Case manager	7
Assistant head nurse	1
Clinical nurse specialist	2
**Years of clinical experience**	
6–10 years	2
11–15 years	7
16 years and over	1
**Work setting**	
Psychiatric/Mental health hospital-based services	3
Addiction treatment centres	2
Primary mental healthcare services	4
Community-based healthcare services for high-risk populations	1
**Area of practice**	
Urban/Suburban	5
Rural/Remote	4
Mixed	1^b^
**Registration year in the program**	
2018–2019 curriculum	6^c^
2019–2020 curriculum	4
**Mean number of session attendance**	9.1 (4.5)^d^
**Session attendance**	
0–5 sessions	3
6–10 sessions	4
11–15 sessions	2
16–20 sessions	1

*n* number, *SD* Standard Deviation

^a^Minimum–maximum = 35.0–46.0

^b^One nurse worked in a large area of the province that included both urban and rural settings

^c^Of those six nurses, two participated in the 2019–2020 curriculum as a second registration in the programme

^d^For first year of participation in the programme

### Data collection

Individual semi-structured interviews with voluntary nurses were held between May 2020 and August 2020 via the Zoom platform (©2021, Zoom Video Communications Inc). All interviews were conducted by the primary author and lasted between 45 and 90 minutes. To facilitate a consistent flow to the participants’ responses, a semi-structured interview guide was developed based on topics generated from the study aim and the research question (Additional file 2). The interview schedule format was structured into four main sections, with prompts and open-ended questions that encouraged participants to share and elaborate on their ideas. Probes, clarification, and paraphrasing were used during the interviews to explore key topics. The interview guide was developed with sufficient flexibility to allow the introduction of new or unanticipated topics. At the end of each interview, participants had the opportunity to further emphasise any issue of importance that had not been adequately addressed and/or to provide additional information. Interviews were recorded using the Zoom platform, after obtaining each participant’s permission, and only audio-based files were safely stored on a secured server at the quaternary academic hospital centre. To enhance the study’s internal consistency, the primary author used a personal journal for compiling insights, decisions, hunches, and important ideas, as the research progressed.

### Data analysis

The full verbatim transcripts of each interview and the primary author’s field notes were both collated to perform a qualitative analysis. A thematic analysis [45] was performed in coherence with the ID methodology to allow the examination of participants’ multiples perspectives, to highlight patterns and discrepancies, and to generate unanticipated insights. Data collection and analysis took place concurrently, with each of them informing the other in an iterative process that allowed reflection and inductive knowledge generation [37]. The analytical process began after a first interview was conducted and extended up to a nine-month period, which enabled a prolonged and close contact with the data.

Each interview was first transcribed by a researcher assistant and then reviewed by the primary author to verify the transcripts’ accuracy with the audio recordings and become familiar with the content. All interview transcripts were subsequently imported into the MAXQDA qualitative analysis software, version 2020.1 (©1995–2020, MAXQDA – Distribution by VERBI GmbH), which later facilitated the coding and management of the data within and across all transcripts. The analysis method consisted of first scrutinizing each interview transcript for meaningful units and progressively organising data into segments of similar experiences and perceptions. Progressive data coding—starting with broad-based categories and then to narrowing the information down into potentially thematic groups—was used to avoid any premature interpretations and precipitate the formation of data patterns. Then, thematic groups were compared across interviews to identify relationships. As relationships between thematic groups became apparent, a hierarchy of themes and sub-themes was created to pinpoints these relationships.

To stimulate further insight into the emerging themes, regular discussion sessions were held between the four members of the research team (GC, JC, JP and DJA) who hold different interdisciplinary backgrounds and complementary research expertise in the fields of health technologies, nursing education and competency development, and concurrent disorders. In addition to investigator triangulation, a member-checking technique was performed [46], thus allowed the research team to discuss and question the accuracy and the resonance of the preliminary findings with the participants’ experiences and perceptions. This technique consisted of emailing all the interviewed participants an electronic summary of the study’s preliminary findings, including a brief description for each theme and sub-theme. Participants were then invited to comment these preliminary findings in the electronic document and send their feedback to the primary author. To guide the participants and gather their reflections and insights, they were asked: “Does this theme match your experience?” and “Do you want to change or add anything?.” Following a deep review of participant feedback (*n =* 6/10), themes and sub-themes were refined and renamed until a consensus was reached between the research team members. For the purpose of this article, the themes, sub-themes and direct quotations from participants were translated from French into English by a certified translator.

### Ethical considerations

This study was approved by the Ethics Committee of the Université de Montréal Hospital Center (#19.295) and the Université de Montréal Research Ethics Committee in Sciences and Health (CERSES-20–017 R). Before each interview, participants were provided with verbal and written information about the nature of the study and informed of the voluntary nature of their participation. All participants provided written informed consent. Interviews were conducted outside working hours, and participants were offered a $CA50 voucher for their contribution. Confidentiality and anonymity were maintained throughout data collection and analysis; no identifiable material was included in any aspect of the research. The primary author was familiar with the ECHO-CD programme, but had no previous relationship with the participants or any professional involvement in the delivery of the educational activities.

## Results

**Fig. 1:**
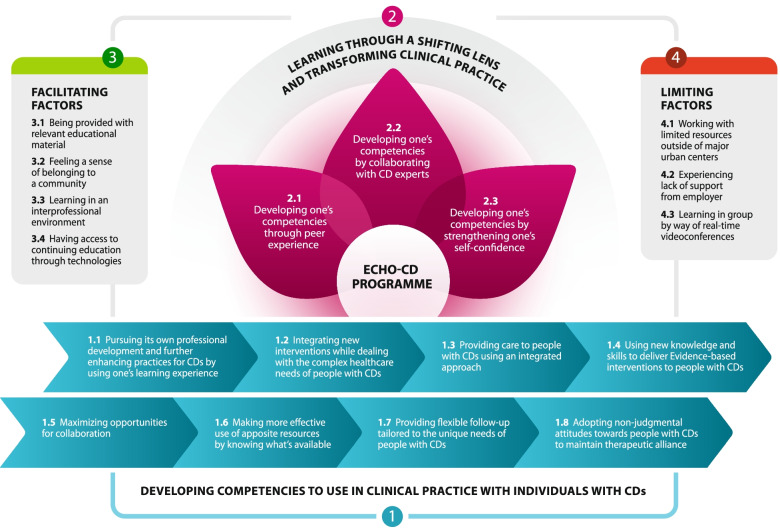
Overview and relationships between themes and sub-themes reflecting the experiences and perceptions of the nurses who participated in ECHO-CD

In total, four distinct yet interrelated themes were identified from the thematic analysis. The four themes with their corresponding sub-themes are embedded in Fig. 1, highlighting how each intertwines to answer the research question.

The first identified theme and its eight contributing sub-themes reflect the progresses nurses made on some clinical nursing competencies and how these competencies have been implemented in their clinical practice with individuals with CDs. The second theme, comprising three sub-themes, depicts the learning process by which the nurses’ experience in ECHO was conducive to the development and implementation of their competencies. Together, the third and fourth themes describe the factors that influenced nurses’ competency development and practice change. Table 3 outlines eloquent excerpts from the interview transcripts for each theme and sub-theme.

**Table 3:** Emerging themes and sub-themes with supporting excerpts from the interview transcripts

Themes	Sub-themes	Excerpts from the interview transcripts
**Theme 1:** **Developing competencies to use in clinical practice when encountering people with CDs**	1.1 Pursuing its own professional development and further enhancing practices for CDs by using one’s learning experience	*- Things we’ve seen or had access to in the ECHO programme, well, I share them. There’s also a moment in the week where my colleagues and I take the time for a little wrap-up… you add an item to the agenda to tell others about an article or whatever, something that caught our attention. That’s something we’re trying to do also now more and more So it’s not ECHO, but we do it on a small scale. (P4).* *- What I appreciated was when we had to fill out the knowledge questionnaires after the session and it showed our weaknesses, the things that we had to work on and improve, like, for me it was alcohol withdrawal and suicide risk. It’s an opportunity for self-reflection. (P5).*
	1.2 Integrating new interventions while dealing with the complex healthcare needs of people with CDs	*- What I realized is that my patients aren’t much different from other patients. So, in many cases I told myself: “Well, don’t lose hope.” There was a patient that it’s been years he’s like the same person doing the same thing the same way, but it was still worth a shot to invest in him. And I think that what I learned over the course of many ECHO sessions is that yes, it’s true, it’s difficult to take care of people with concurrent disorders, but despite everything there are still solutions and things that you can do. (P4).*
	1.3 Providing care to people with CDs using an integrated approach	*- However, I find in fact that during ECHO … it was nonetheless at the heart of the matter to work as much on physical health, as on mental health, as on substance use disorders. I think that it was something that kept coming back at just about all the ECHO sessions, the importance to address all of the different issues at the same time. There was also a didactic presentation that talked a little about the effects of substance use on, among other things, on cognition. Being able to assess the impact of withdrawal and to adapt ultimately our interventions to the clinical situation. (P10).*
	1.4 Using new knowledge and skills to deliver evidence-based interventions to people with CDs	*- I have the impression that since ECHO, I am more developing motivational interviewing in my practice. Of course I was already doing it, I already had some basic knowledge, but I have the impression that it’s something that’s now a little more developed. I think that I communicate it better through my practice, especially if the patient really wants to hear it, you know. (P1).*
	1.5 Maximizing opportunities for collaboration	*- The more contact I had with addiction services’ team, the easier the communication with them, the more I know their services, the better I can then explain them to our patients and inform them adequately, in the end, on what’s available and how to access those services. (P1).* *- My colleagues and I we help each other quite a bit and we form a pretty tight-knit team, so when we’re faced with challenges, well, we look for other ideas, other resources in our team. We try not to go it alone when there are complex situations. So this way we feel less overwhelmed. (P5).*
	1.6 Making more effective use of apposite resources by knowing what’s available	*- Like it or not, it helps to do some mentoring like ECHO with various types of professionals and organizations. So that incites my team and I even more to use existing services for further information and clinical support. It opens things up. (P9).*
	1.7 Providing flexible follow-up tailored to the unique needs of people with CDs	*- I think I am less trying to rush things up. I’m asking if the time is right for the person to quit alcohol and really take the time to patch things up properly afterwards. Sometimes it’s a matter of paving the way better, maybe take a little more time at first to prepare the person to quit for it to be more effective in the long run. (P2).* *- Having the treatment plan up to date… But sometimes you don’t always have the time, so you don’t always do it. Often, it’s the first thing that falls by the wayside. But realizing that in fact, well, it’s important to question things regularly, to update them all the time. So that was highlighted during ECHO. (P4).*
	1.8 Adopting non-judgmental attitudes towards people with CDs to maintain therapeutic alliance	*- I have the impression that I’m more understanding … much less judgmental. I imagine that it must transpire in my body language that I am not the least bit judgmental and all I really want to do is properly assess the situation and direct the person towards the proper resources, guide them properly. (P9).*
**Theme 2:** **Learning through a shifting lens and transforming clinical practice**	2.1 Developing one’s competencies through peer experience	*- Me personally, I think, it’s really a matter of give and take. There is an important aspect of sharing … sharing experiences, challenges. Like for me, when I presented a case, well it was quite confronting, but still, I benefited from many recommendations and ideas. And you know, I think it could have been useful to others as well. (P4).*
	2.2 Developing one’s competencies by collaborating with CD experts	*- There were a lot of cases of schizophrenia, complex situations, who were isolated and what to do to mobilize them again. That, I found that interesting. I remember one time when I told myself: “Hey, I myself wouldn’t know what to do with that”. And I admired the team’s dedication and how they approached that. I really would have needed support if I had been in their situation. Having a vision of loads of mentors from across the region, in the end …I found that to be a rich source of information. (P6).*
	2.3 Developing one’s competencies by strengthening one’s self-confidence	*- Sometimes, you feel like … you’re not good at what you do or you question your abilities a lot precisely when things fail to come to a successful conclusion or you keep going through the same problems with some patients over and over, and to share this with others from regions other than our own … for those of us who do not work in the major urban centers with specialists… So that, too, is reassuring. It’s to see that, in the end, what we manage to do with the means at our disposal, well it’s not bad at all. (P4).*
**Theme 3:** **Factors facilitating competency development and practice change**	3.1 Being provided with relevant educational material	*- Personally, what I liked a lot was the didactic presentations. And what’s good is that they’re all backed up with references and they’re listed on the website. That’s super interesting because I went and retrieved a few of them. So, what it allows us to do is to base our interventions then on the literature. (P3).*
	3.2 Feeling a sense of belonging to a community	*- You know, you feel a little like you’re not all alone. At times you have questions, and you don’t know who to turn to. So, this (ECHO-CD) was the perfect place to do so. (P2).*
	3.3 Learning in an interprofessional environment	*- In the ECHO sessions on concurrent disorders, well, the panel (team of experts) is interdisciplinary. That, in my opinion, is a winning ingredient there, precisely because our clientele is so varied, so complex and multidimensional. (P3).*
	3.4 Having access to continuing education through technologies	*- What I like about ECHO is the easy access. First, the fact that it’s free makes it accessible to everyone. And then, the fact that the sessions are delivered on Zoom, well, personally I found it helpful to be able to see the people, to be able to discuss things easily. (P8).*
**Theme 4:** **Factors limiting competency development and practice change**	4.1 Working with limited resources outside of major urban centers	*- I would have liked for our own physicians to be involved more in ECHO, like for them to be more present to be able to gain a greater awareness of what’s going on elsewhere and like stimulate their imagination. It would have been more interesting for us afterwards to put what we learned into practice. Because, as it turns out, sure, there were nice proposals made during ECHO, but… then, I did not have anyone to back me up about trying new treatment options. Because there are a lot of medical decisions to be made as well behind it all. (P5).*
	4.2 Experiencing lack of support from employer	*- Well, what worked against me is that I’ve become a head nurse assistant along the way. That’s why I couldn’t put things into practice or integrate them as much and to try new things out with my patients because … I was really pulled out of that role. (P1).* *- What I retained was, I was very passive, in the sense that, I didn’t contribute any case-based discussion. Plus, it took place at a time where I was pretty much alone in my team with a novice nurse, so I didn’t have the time to prepare any cases to present for ECHO. I could have been more assiduous. (P2).* *- The computer I was using at work didn’t have a webcam, so I was only able to chat and listen. (P7).*
	4.3 Learning in group by way of real-time videoconferences	*- For sure, I was very much questioning myself in the first sessions. So, when the time came to make recommendations to other participants, well, I had some reservations … I would tell myself: “Well, maybe my vision isn’t necessarily the right one.” (P4)* *- We were, my team and I, on one computer, so there was one person in front of the computer, and others in front of a large screen. Consequently, participation wasn’t like optimal, to be able interact, I mean. So, generally, it was more through chat that we’d say: “Write this.” But, at times, the time it took to write that, well, we’d moved on to something else. (P5).*

*ECHO* Extension for Community Healthcare Outcomes, *P* Participant

### Theme 1: Developing competencies to use in clinical practice when encountering people with CDs

The findings showed that nurses who participated in ECHO-CD have progressed in eight clinical nursing elements of competencies. Above all eight progressions, nurses often indicated that ECHO-CD reinforced their willingness to pursue their own professional development and their involvement in advancing work practices in the field of CDs (sub-theme 1.1). Nurses were able to reflect on their own practice and identify their strengths and learning needs in CDs. They viewed ECHO-CD as a first step toward obtaining further clinical guidance and/or refining their expertise in CDs. Nurses felt more aware of how to update their current knowledge in CDs and to incorporate that into their own practice, as some of them accessed a variety of other formal and informal learning opportunities after ECHO-CD. Also, nurses provided their colleagues with assistance regarding CDs by sharing their new learning acquisitions.

A second perceived progress was to better manage the complex healthcare needs of people with CDs (sub-theme 1.2). Nurses recognized that positive change in people with CDs can be difficult but is not impossible. ECHO-CD helped them to develop and maintain a sense of hope towards people with CDs and to channel this sense into their care provision. Nurses were better managing healthcare needs by being able to adapt their professional ideals to their patients’ needs, while seeking for help when they experienced feelings of helplessness. It was also noted that nurses felt more comfortable dealing with unexpected situations and were further inclined to embrace ambiguity towards decision making. This is illustrated in the following quotes:

What I realized is that positive changes are possible for people with concurrent disorders. […] I thought so before, but, with ECHO, we were able to find concrete solutions to deal with complex clinical situations and we saw that recovery really is possible. Perhaps not a full recovery, but an improvement of the person’s condition.”(P3)

“I can say that I’m more comfortable working with concurrent disorders than I used to be. I feel more confident in my ability to treat patients, instead of referring them right off to other services or to a specialized service.” (P2)

Nurses considered that integrating mental health and addiction care was an important aspect of their professional role, and that ECHO-CD allowed them to further incorporate this fundamental CD-value into their clinical practice (sub-theme 1.3). Nurses indicated they felt more equipped to intervene on both conditions simultaneously by performing a comprehensive assessment of health and social needs and establishing priorities into their care plan. In addition, nurses mentioned that their participation in ECHO-CD allowed them to further emphasise on interprofessional collaboration when developing or reviewing their patients’ care plan, by sharing responsibilities and regularly communicate with coworkers, other healthcare professionals, and/or community health workers from various organisations (sub-theme 1.5).

Nurses also perceived they had acquired new CD-related knowledge (sub-theme 1.4), and information about where people with CDs can access more in-depth advice and/or services (sub-theme 1.6). These new learning acquisitions were somehow different for each nurse, according to their academic and professional background or work setting. For instance, nurses who worked in mental health settings gained knowledge about addiction treatments, and skills in withdrawal management and motivational interviewing, while nurses in addiction treatment centres learned about distinguishing primary versus induced disorders, dealing with personality and anxiety disorders, and psychiatric medication. As one mental health nurse said:

“During ECHO, we talked about safer injecting, best practices for harm reduction, and how we really can empower patients to inject more safely. I learned about what signs and symptoms to look for when my patients aren’t doing so well, and what strategies I can use [to help them].” (P10)

During the interviews, nurses reflected that their care approach had become more flexible to the unique needs of their patients (sub-theme 1.7) as a result of their increased empathy towards their patients’ choices and lifestyle. Lastly, nurses noted that their participation in ECHO-CD helped them gain a better understanding of the profound challenges individuals with CDs face in their life. This opportunity led the nurses to reflect on their own personal beliefs and/or misconceptions, a process that, in turn, facilitated the adoption of non-judgemental attitudes towards CDs (sub-theme 1.8). As this nurse explained:

I know [ECHO-CD] helped me work through my misconceptions. […] I understand a little better what can make people to use [substances] and just how difficult it is to quit. It’s not that they don’t have willpower and […] well, you know, that it’s just not easy to overcome a substance use disorder.” (P1)

### Theme 2: Learning through a shifting lens and transforming clinical practice

Theme 2 enhances our understanding of how ECHO-CD—in terms of learning methods and strategies—produced meaningful learning experiences from the nurse perspective. According to nurses’ perceptions, social interaction was a key component of ECHO-CD as it allowed them to learn from their peers’ experience, and to sometimes share their own suggestions or ideas (sub-theme 2.1). Nurses specifically valued the case-based discussion period, in which clinical story telling helped them to feel less alone by normalizing the difficulties they faced when caring for individuals with CDs. Even for those nurses who did not present a clinical situation, listening to authentic and rich clinical experiences from peers led them to reflect on their own learning and practice, which put them in a favourable position for self-growth. Two nurses expressed it like this:

For me, it’s really about learning from others’ expertise by having the opportunity to hear participants’ questions and what others would then propose. We often ask ourselves the same questions. It makes you realize: “Oh yeah, maybe I could do such and such with my patient, too.” As a professional, I found that extremely enriching. (P3)

We see that others are having very similar experiences, so in the end it comforts us in what we’re doing […] In a way, it’s comforting to know there’s hope. We compare ourselves with others but then we realize that everyone is facing the same challenges. So, by comparing ourselves with others, we realize we aren’t to the only ones with these issues. It’s reassuring and reinforces our practice.” (P7)

Nurses appreciated benefiting from the recommendations and feedback of an interprofessional team of experts in CDs (sub-theme 2.2). Nurses viewed this opportunity as immensely important as they received support from experts with whom they would otherwise not be able to connect with on a regular basis. A key finding was that nurses viewed these healthcare professionals as mentors. Nurses felt more inclined to adapt their clinical practice based on the experts’ recommendations since they valued and recognized their expertise in CDs.

Nurses’ learning experience during ECHO-CD helped them to strengthen their confidence in caring for people with CDs (sub-theme 2.3). This sense of confidence was progressively internalized as nurses went through the programme curriculum. For some nurses, receiving recommendations and feedback from experts reassured them that their clinical practice aligned with CD evidence-based practice. For other nurses, listening to their peer’s experiences allowed them to notice the benefits of changing some of their current behaviours. A one nurse explained:

Sometimes we have ideas [that are outside of the box] and we think: “Are we completely off track here?” But, when we see what others are doing, it’s like “Well, okay. If it works for them, it should work out fine for me, too.” (P5)

The thematic analysis also provided insights into the relationships between theme 1 and 2—meaning which educational components of the programme most contributed to the respective progression of each clinical nursing competency. For example, when “integrating new interventions while dealing with the complex healthcare needs of people with CDs” (sub-theme 1.2), nurses developed their clinical judgment competency further by being exposed to the realities of their peers and by observing others approaches to challenging clinical situations (sub-theme 2.1). When a clinical situation was presented and then discussed, nurses felt reassured that others in the same position might experience the same struggles. Similarly, most nurses gained scientific and up-to-date knowledge in CDs (sub-theme 1.4) from receiving guidance from experts (sub-theme 2.2).

### Theme 3: Factors facilitating competency development and practice change

Nurses greatly appreciated the material provided during the programme (sub-theme 3.1). There was a consensus among the nurses that the didactic presentations’ content was relevant and could be easily translated into their practice. Reflecting on their overall learning experience, nurses felt a genuine sense of belonging to a community (sub-theme 3.2), which allowed them to establish trust towards the group participants and maintain their engagement into the programme. Nurses reported that ECHO-CD fostered an open dialogue providing opportunities to communicate emotions of distress without feeling judged by others. This convivial atmosphere empowered by the experts and the facilitator’s active hosting were perceived as positive contributors to learning. These perceptions are illustrated in the following quote:

Everyone was expressing their point of view, sharing, bringing a different perspective […] Personally, I found there was a nice sense of camaraderie that made everyone feel very comfortable about asking questions and getting answers. Everyone was very respectful when others spoke and waited their turn to speak. [The team of experts] made sure there was enough time for people to answer questions. Because of this, I found it was really friendly and fun. (P9)

Based on the nurses’ perceptions, one important strength about ECHO-CD was the interprofessional approach that generated enriching discussions (sub-theme 3.3). This group wisdom allowed for meaningful sharing of information and strengthened the acquisition of cross-disciplinary knowledge and skills in CDs, rather than focusing on silos of discipline-encapsulated knowledge. As such, being exposed to a diversity of allied and medical professions allowed nurses to further use an integrated approach to mental health and substance use issues (sub-theme 1.2), and foster collaboration between teams and agencies (sub-theme 1.5). One nurse said:

I really liked it a lot because the panel was diverse. It wasn’t just physicians’ point of view or pharmacists’ point of view […] The diversity and getting to hear everyone’s expertise made it interesting. There were a lot of different ideas and suggestions. It was enriching because we don’t have any occupational therapists in my team, so that opened me to new approaches in my practice.” (P8)

### Theme 4: Factors limiting competency development and practice change

Three sub-themes were identified as factors that negatively influenced the development and implementation of the nurses’ competencies, in relation to both the educational and the clinical settings. Firstly, an uneven distribution of appropriate resources for CDs was commonly reported by nurses as an important factor that precluded their capacity to provide adequate and flexible care (sub-theme 4.1). This situation was particularly exacerbated for nurses working outside of large urban centres, where limited specialized resources are available. This finding came from witnessing that many nurses struggled to work in a health care system that seeks to avoid people with CDs, and that they often experienced a lack of appropriate referral pathways. As such, navigating through the multiple layers of the healthcare system to ensure care coordination was described by nurses as complex and demanding. During ECHO-CD, some nurses also claimed that expert recommendations about CD resources did not fit well with their local context of practice:

Since I’m not in a major urban centre, I don’t have access to all resources. [During] the case discussions in the ECHO programme, [the team of experts] would propose such and such a resource to help patients. But I don’t have [access to] the same array of services as in urban centres. So, I think you have to be creative and still try to provide services tailored to your patients’ needs. (P6)

Within the context of limited human resources, nurses also expressed they were often in the position of arguing against their coworkers about the best approaches to CD care. Most of the time, these situations took place when nurses sought to incorporate new ideas into their patients’ care plan, based on what they had learned in ECHO-CD. Unfortunately, nurses had to demonstrate to their coworkers that their suggestions were scientifically proven, and how these new ideas would constitute an added value to the current care plan. Many nurses also felt that advancing practices in the field of CDs would require “philosophical change”, noting that most healthcare professionals continue to work with a punitive approach with patients with CDs.

During the interviews, nurses felt that their participation in ECHO-CD was not always optimal and that further support from their organizations could have facilitated their engagement and learning (sub-theme 4.2). Two nurses expressed it like this:

Connectivity was a problem […] at our hospital. We couldn’t get an internet connection, so I had to use my cellphone or work from home. I mean of course, I was able to connect at home, but at work, they wouldn’t let me. So that was a major issue. (P3).

[ECHO-CD] lasted an hour, an hour and a half, so I couldn’t always attend to the last didactic part because I had other things to do, like appointments with patients. (P5).

While nurses had a positive regard about learning from their peers’ experience, they also identified the group modality as a major downside (sub-theme 4.3). Nurses expressed apprehensions and a sense of fear when beginning the programme, which were triggered by feeling intimidated from others’ expertise in CDs, as expressed in the following quote:

There were lots of people during ECHO, a heck of a lot. Personally, I was really impressed, it was super interesting. It was a little intimidating too, though. At first, the idea of presenting a clinical situation was intimidating […] because it was online in front of a lot of people, like a lot of people. You don’t know who they are […] it’s not like in a classroom, where after two or three times you feel a little more at ease. (P1)

Nurses were particularly reluctant to use the video feature of the videoconference technology for presenting a clinical situation or when they believed their knowledge or experiences would have benefit the group. To overcome this, nurses felt more comfortable using the chat forum to ask questions and share their ideas. Nurses noted, however, that their apprehensions were allayed by the presence of a facilitator, and once they received constructive feedback and encouragements by the experts.

## Discussion

### Main findings

Guided by a socio-constructivist perspective, this study aimed to explore the experiences and perceptions of the nurses who participated in an ECHO programme for CD management about the competencies they developed and used in their clinical practice, and the factors that influenced this process. Our findings support that an interprofessional, videoconference-based educational programme on CDs reinforces the development of nurses’ competencies and clinical practice, through interactive learning with peers and experts, and through self-reflection, and within an environment that can be either facilitating or limiting. These findings are congruent with the results of the scoping review by Garrod’s et al. [6] that conclude that continuing education was an effective means of improving attitude, increasing knowledge and confidence, and supporting practice change in nurses who provide care to people with CDs. In complementarity with prior studies of ECHO programmes in the field of CDs that reported broad statistical results on participants’ knowledge and confidence gain [22, 28–32], this study provided rich data on the manifestations of the development of clinical nursing competencies, and an in-depth understanding of how this process took place, and what factors influenced it.

This study highlighted that ECHO-CD created an educational space allowing nurses to further develop and use their competencies in their clinical practice with individuals with CDs. These findings closely correlate with essential competencies established in shared-competency frameworks in CDs [47], interprofessional guidelines of CD evidence-based interventions [5, 11], and Canadian standards of practice within the field of mental health nursing [48]. Compared to these competency-based frameworks, our results indicate that nurses progressed from a “core” to a “generalist” skill level in working with individuals with CDs, for most of their clinical nursing competencies. As opposed to this trend in their progression, our results indicate that some nurses evolved to a “specialist” level in their professional development, as they were able to critically analyse their own practice and assist colleagues in reviewing their knowledge, skills, and practice in relation to CDs.

As indicated in previous studies [8, 49], the nurse participants viewed their clinical practice with people with CDs as a constant struggle between their professional ideals and their patients’ expectations and choices. Even though most nurses stated feeling emotionally exhausted when caring for more complex CD situations, participating in ECHO-CD allowed them to cultivate hope and maintain therapeutic optimism. Hence, ECHO was not only conducive to acquiring new knowledge or learning, but it has also fostered a supportive environment that was essential to nurses’ well-being. Similarly, Petrakis et al. [50] conducted a systematic review regarding educational approaches to leveraging CD competencies and found that supervision supported healthcare professionals in difficult situations by helping them to reflect on the learning process that occurred.

We found that during ECHO-CD, nurses made some progress in their clinical competencies by interacting with peers, being mentored by experts, and building their own confidence—all of which are linked to the three key theoretical foundations of the ECHO model, namely Wenger’s Theory of Communities of Practice [43], Lave and Wenger’s Situated Learning Theory [42], and Bandura’s Social Cognitive Theory of Behavioral Change [41]. This finding reiterates the results of a previous qualitative study on primary care providers’ experiences of participating in an ECHO programme on resistant hypertension [51]. In that study, participants illustrated the benefits of each theory’s educational principles. In this study, we have extended those findings by emphasising on how each of the learning theories used in ECHO-CD were intimately related to the distinctive progression of certain competency elements.

In terms of facilitating factors, most nurses felt that the didactic presentations were crucial to acquiring specific and up-to-date knowledge on CDs, and they would appreciate that more time would have been allocated to this. This finding was not anticipated, given the fact that traditional lectures have been documented as less effective than active learning. While published work within the field of nursing education has called for alternatives to traditional continuing education [52], our study findings showed that nurses rewarded ECHO-CD for its capacity to join both active and passive methods of learning. One possible explanation is that nurses may need more guidance or direct supervision in order to further develop their competencies in CDs [6]. Indeed, entering an interdisciplinary, videoconference-based environment with unknown colleagues and experts triggered uncertainties and questioning in nurses about how they provided—or should provide— care to people with CDs.

Considering that the ECHO model was originally designed for medical providers only, an interesting finding from this study is that nurses viewed the interprofessional environment as a key factor that enabled their competency development. The positive impacts of interprofessional education on knowledge and skills in collaboration are well documented within nursing education research [53, 54], as well as in the field of mental health nursing practice [55]. Similarly, studies within the ECHO literature have outlined several benefits of interprofessional education from the experiences and views of participants such as an increased awareness of one’s and colleagues’ professional role and open-mind attitudes towards interprofessional collaboration [56–61].

While technical issues such as Internet connectivity and bandwidth have been reported as important barriers to participation in other ECHO programmes worldwide [61–63], this study on the contrary revealed that nurses had a positive experience with the videoconference technology. The Zoom software was viewed as a convenient and flexible means of learning that does not require advanced technological skills. This finding is consistent with other authors, such as Gagnon et al. [64] who concluded in a systematic review that ease of use and perceived usefulness were the most common factors influencing the adoption of information and communication technologies by healthcare professionals.

Regarding limiting factors, our results show that diligent participation was a challenge for nurses, mostly due to the lack of support from their employer and/or organization. Barriers to participation included time/practice-related issues, lack of appropriate technological material to fully take part in the programme, increases in nursing workload and rolling staff placement. These findings are congruent with existing literature on virtual communities of practice in other contexts than ECHO. For instance, an integrative review by McLoughlin et al. [65] revealed that a large proportion of participants function in the role of “lurkers” (i.e., participants who do not actively participate), especially when participation is neither required nor requested by the community. Nevertheless, it has been suggested that “lurking” can be a first step for participants who are apprehensive about using technology or who lack confidence or experience.

Overall, this study builds on growing literature describing factors that can positively and negatively influence learning within continuing professional education [66]. It is novel in that it exposes—from nurses’ experiences and perceptions—that such factors are multi-faceted, ranging from proximal (i.e., personal and educational factors) to distal influences (i.e., contextual factors such as social, cultural, environmental, organizational and political influences). Hence, strategies for fostering competency development and practice change in nurses will need to address a broad range of factors simultaneously. Interestingly, our findings indicated that personal and educational factors (i.e., educational material, sense of belonging to a community, interprofessional environment, videoconference technology, group learning mode) identified by nurses mirrored most of the barriers and facilitators previously described in a broad spectrum of the ECHO literature (e.g., chronic pain, Hepatitis C, paediatric care) [17, 58, 62, 67], while contextual factors (i.e., limited resources, lack of employee support) specifically accounted for evidence from ECHO programmes in the field of mental health/psychiatric and addiction care [28, 32, 68–70].

### Limitations

This study has some limitation. First, 32 nurses from the whole potential study population (*N* = 65) met the inclusion criteria and, of these, 10 participated in this study. Therefore, a selection bias may exist since the nurses who did not participate in this study may have different experiences or perceptions regarding ECHO-CD. However, the data collection strategies used in this study, which included ensuring a certain level of heterogeneity between the characteristics of the recruited participants and conducting in-depth interviews, helped strengthen the credibility of the findings.

To mitigate the impact of the COVID-19 pandemic and to encourage remote participation, the Zoom platform was chosen as a convenient alternative to in-person interviews. A downside of this digital option is that technical issues can unexpectedly arise, and these can adversely affect the quality of the interviews and hinder the participant’s spontaneous interactions [71]. Although Internet connectivity was not a particular issue in this study, visual cues, such as non-verbal reactions, were more difficult to capture since the camera only showed the participants’ upper body. However, given that this study’s phenomenon of interest is a professional, non-sensitive topic (i.e., nurses’ competency development within the context of a videoconferencing educational programme), using videoconference technology was deemed to be an appropriate means for conducting the interviews with the nurses. In addition, during participation in ECHO-CD, the nurses interviewed in this study were comfortable using the technology and knowledgeable about its functionalities, due to their previous experience with the Zoom platform.

Our results should also be interpreted with caution because this study included nurses from a single ECHO programme and from just one province. As such, a detailed description of the educational programme was provided (Table 1) to facilitate the replication of the intervention and its adaptation in other contexts. Lastly, we acknowledge that our findings may not be indicative of everyone’s experience, including other health professions or local contexts that may differ.

### Implication of the findings for education, clinical practice, and research

Drawing from this study’s findings and existing research evidence, we have underpinned in Table 4 a number of recommendations which are likely to be beneficial for education, clinical nursing practice, and research.

**Table 4:** Recommendations for education, clinical practice, and future research, based on the study findings

Key findings	Recommendations
**Education**	
- This study emphasizes the pressing need for all nurses working with people with CDs to be offered basic CD training—even the experienced nurses, who may benefit from continuing education opportunities to expand their scope of practice.	- Both mental health/psychiatric and addiction issues must be covered in undergraduate nursing programs [48]. - Continuing professional education in CDs should be standardized within the clinical settings.
- ECHO is an online collaborative model of continuing education that relies on active participation for content.	- Limited and fragmented participation should be addressed in future ECHO programmes—or in other types of online collaborative learning models—by developing mechanisms for engaging non-contributing participants with active knowledge sharers.
- Despite its many benefits, group learning can negatively influence participation. - Nurses may not feel comfortable sharing with the group or interacting with other participants, especially with those in a senior position or with more expertise.	- Ensuring that educational programmes have a positive, encouraging environment can help to build a culture of trust between participants. - Carefully selecting facilitators who are both knowledgeable in the specialist area and team builders in their approach to sharing knowledge. - The key task of the facilitator is to create a safe learning environment in which participants can share both their successes and their challenges.
- ECHO—and other types of videoconference-based educational programmes—depends on a reliable Internet connection and the use of visual connectivity to improve communication and relationship building between participants.	- Access to a dedicated technological support service during the sessions is a prerequisite to ensure the successful conduct of the learning activities.
**Clinical nursing practice**	
- Providing optimal care for individuals with CDs is challenging for nurses, and it has been associated with a higher vulnerability to burnout syndrome, and low job satisfaction and work engagement among nurses [72, 73].	- Mentoring programmes and clinical supervision should be further incorporated into nursing practice and clinical settings. - A team-based approach to continuing professional development should be prioritized to enhance collaboration and communication between colleagues, and to align care practices around shared values and goals. - Emotional support is essential to enrich the continuing education programmes already in place.
	- Future continuing education programmes should integrate self-confidence enhancing strategies to support nurses in caring for individuals with complex healthcare needs.
- The factors influencing nursing competency development are multi-faceted.	- A tailored approach to continuing professional education, in which the structure and clinical content of interventions are personalized to the needs of participants is essential to facilitating and sustaining changes in clinical practice. - Researchers, educators, and clinical leaders should develop mechanisms to reinforce nurses’ participation in and motivation towards continuing professional education, and should do so by engaging them in all design and maintenance procedures, from planning their initiatives to evaluating and improving them. - Protected time periods during working hours should be established for nurses so that they may benefit from continuing professional development opportunities.
	- Working environments should provide nurses with the minimum requirements of technical equipment (e.g., desktop or laptop computer, Internet connection, webcam or HD cam, and speakers and microphones), so that participants can fully benefit from online educational programmes.
	- Supportive leadership from local stakeholders (e.g., care coordinator, health administrators, organizational leaders) is crucial to fostering best care practices and promoting a culture of change.
**Future research**	
- Future research and evaluation are needed to extend our current understanding of the barriers to and the enablers of engagement in ECHO. For example, subsequent studies should examine what level or type of engagement is ideal for learning to occur and to be sustained in longer term outcomes.	
- Given that the ECHO model allows developers to adapt its content and structure to local needs, further research is needed to better understand how variations in the educational intervention may affect participants’ learning and clinical practice.	
- More research also needs to be conducted on the effectiveness of ECHO for increasing learning and professional performance. For example, studies should focus on answering the following question: What are the best educational practices for using ECHO, and what areas should be improved to enhance its effectiveness in supporting competency development and in sustaining changes in clinical practice?	
- Finally, further research should aim to examine interaction processes in educational interventions that simultaneously use many learning methods, and further investigate their impact on nurses’ learning and practice-level outcomes.	

*CDs* Concurrent disorders, *ECHO* Extension for Community Healthcare Outcomes, *HD* High definition

## Conclusions

To our knowledge, this is the first study of an ECHO programme for CD management aiming to explore how nurses further develop and implement their competencies in their clinical practice. Building on a socio-constructivist perspective of learning, this study stands out for its’ philosophical and conceptual originality, and sheds new light on the ECHO model contributions to the competency development and clinical practice of nurses. It was unique in that it sought insight into the progression of nurses’ learning while gathering rich data about the factors that facilitated or limited this process. This study therefore contributes to the current body of knowledge on the ECHO model and adds value in terms of increased understanding of what are the optimal educational methods and learning environment that can support nurses in developing high-level of competencies in CDs. It also adds some support to previous research emphasizing the current need for nurses to participate in formal opportunities for continuing professional development over the span of their careers.

Our results suggest that ECHO-CD offered an efficient and convenient alternative to traditional continuing education, since it situates learning within authentic professional practice using videoconference technology. Furthermore, ECHO-CD was an opportunity for experiential learning that helps nurses apply their new knowledge to improving their problem solving and to meeting the dynamic and complex needs of patients with CDs. However, to facilitate the engagement and learning of local participants, this type of educational intervention must be flexible and sensitive to their specific needs. These findings are critical to both the field of nursing education research and continuing education research in the health professions. They will provide guidance to researchers, educators, nurses, and decision makers who are developing, implementing, evaluating, and escalating future educational interventions in the field of CDs.

## Supplementary Information

**Additional file 1.** Research Checklist

**Additional file 2.** Individual semi-structured interview guide^a^

## Data Availability

The datasets that were generated and/or analysed in the current study are not publicly available due to their sensitive nature (i.e., data collected through individual interviews in the form of personal verbatim), but are available from the corresponding author on reasonable request.
