# Supplementary material for: Experiences and perceptions of nurses participating in an interprofessional, videoconference-based educational programme on concurrent mental health and substance use disorders: a qualitative study
Source: BMC Nurs. 2022 Jul 4;21:177. doi: 10.1186/s12912-022-00943-w (PMC9251915; doi:10.1186/s12912-022-00943-w)
Supplement: Supplementary file 1 — Additional file 1. Research Checklist [file 12912_2022_943_MOESM1_ESM.docx]

Additional file 1. Research Checklist

| **Consolidated criteria for reporting qualitative research (COREQ): a 32-item checklist for interviews and focus group (36)** | | |
| --- | --- | --- |
| **No.** | **Item** | **Description** |
| **Domain 1: Research team and reflexivity** | | |
| ***Personal characteristics*** | | |
| **1** | Interviewer/facilitator | GC (principal investigator) |
| **2** | Credentials | GC – RN, MSc, PhD Candidate  JC – RN, PhD  JP – RN, PhD  LB – RN, PhD  GR – RN, MSc, PhD  DJA – Psychiatrist, MD, MSc, FRCPC |
| **3** | Occupation | GC – PhD Candidate (Faculty of Nursing, Université de Montréal, QC, Canada).  JC – Principal Supervisor of GC’s doctoral research project; Professor (Faculty of Nursing, Université de Montréal, QC, Canada); Researcher and Chairholder (Université de Montréal Hospital Research Center, QC, Canada).  JP – Co-Supervisor of GC’s doctoral research project; Professor (Faculty of Nursing, Université de Montréal, QC, Canada); Scientific Director (FUTUR Team, Faculty of Nursing, Université de Montréal, QC, Canada).  LB – Member of GC’s doctoral thesis committee; Professor (Faculty of Nursing, University of Montreal, QC, Canada).  GR – Postdoctoral Fellow (Institute for Health System Solutions and Virtual Care, Women’s College Hospital, Ontario, Canada); she was involved in the conceptualization of the study (i.e., study research protocol).  DJA – Co-Supervisor of GC’s doctoral research project; MD, Associate Professor (Faculty of Medicine, Université de Montréal, QC, Canada); President (Centre of Excellence and Collaboration in Concurrent Disorders, Université de Montréal Hospital Center, QC, Canada); Clinical Researcher (Université de Montréal Hospital Research Center, QC, Canada). |
| **4** | Gender | Interviewer: female identifying.  Research team: three females identifying and one male identifying. |
| **5** | Experience and training | GC – She has clinical experience and expertise in CDs. She has experience in qualitative, quantitative and mixed methods research. She had a specialized training in mixed methods research during her PhD training. She also has academic experience as a nurse preceptor university lecturer for undergraduate and graduate nursing programmes. She also has previous experience in conducting individual semi-structured interviews.  JC – She has expertise in quantitative research, including research pertaining to the development and evaluation of evidence-based practice interventions for people living with chronic conditions. She also has experience and expertise in health services research and digital health.  JP – She has expertise in qualitative research, including nursing education research and nursing discipline advancement.  LB – She has expertise in qualitative research, including nursing education research and competency development.  GR – She has expertise in implementation science, knowledge translation practice and research, and in the development and evaluation of virtual nursing interventions.  DJA – He is a psychiatrist with a specialization in CDs. He has expertise in conducting registered controlled trials, including pharmacologic and non-pharmacologic interventions for patients with CDs. He was directly involved in the development and implementation of the ECHO-CD programme. |
| ***Relationship with participants*** | | |
| **6** | Relationship established | There was no relationship established with participants prior to the beginning of the study. First contacts with the study participants were established by GC during at recruitment.  Prior to the study conduct, GC assisted to three sessions of the ECHO-CD programme. Her objective was to observe the sessions’ conduct and learning activities, as well as the interactions between participants and experts. She did not interact or intervene with participants during the three sessions. |
| **7** | Participant knowledge of interviewer | All individual semi-structured interviews (*n=10*) were conducted by GC.  During recruitment, GC introduced herself as a PhD candidate at the Faculty of Nursing, Université de Montréal Faculty, and as the principal investigator of the study. All contacts and conversations with participants (e.g., details about the study objectives and its’ implication for participants, taking appointments, follow-up for the member-checking technique) occurred through email, before and after the interviews. Participants were made aware of the interviewer’s academic and clinical background, personal goals, and reasons for doing the research. A summary of the research project was provided to participants during recruitment verbally and in a consent, prior to each interview.  As participant were aware of the interviewer’s presence and role towards the study, a desirability bias may exist. It is possible that some of the participating nurses have overrated the educational intervention’s benefits and/or impact on the development of their competencies and clinical practice. |
| **8** | Interviewer characteristics | GC is a RN since 2012 (license from the *Ordre des Infirmières et Infirmiers du Québec*) and she has clinical experience in mental health/psychiatric and addiction care. She also worked as a clinical nurse specialist in a specialized care unit devoted to individuals suffering from CDs at a quaternary academic hospital centre from 2014 to 2017. She has experience in individual semi-structured interviews with healthcare professionals and patients, including advanced skills in motivational interviewing and psychotherapeutic approaches.  Since 2017, she is a PhD candidate in the field of nursing sciences and graduate student researcher at the Université de Montréal Hospital Research Centre. She was in her fourth year of PhD at the time of data collection and analysis. She conceptualized the study, led the protocol development for this study, and she oversaw each stage of the study conduct, starting from the participants’ recruitment to data collection and analysis. |
| **Domain 2: Study design** | | |
| ***Theoretical framework*** | | |
| **9** | Methodological orientation and theory | Done.  **Methodological orientation:** Interpretive Description approach – See ‘Methods’ section (‘Research design’ subsection) – P.8, lines 197-203.  **Theory:** Socio-constructivist epistemology; holistic, context-bound, and experientially based conception of learning and competency development – See ‘Background’ section (‘Philosophical and conceptual underpinnings’ subsection) section – P.7-8, lines 174-186. |
| ***Participant selection*** | | |
| **10** | Sampling | Done.  See ‘Methods’ section (‘Participants and recruitment’ subsection) – P.9, lines 220-231. |
| **11** | Method of approach | Done.  See ‘Methods’ section (‘Participants and recruitment’ subsection) – P.10, lines 232-239. |
| **12** | Sample size | Done.  See ‘Methods’ section (‘Participants and recruitment’ subsection) – P.10, lines 240. |
| **13** | Non-participation | NA. |
| ***Setting*** | | |
| **14** | Setting of data collection | Done.  See ‘Methods’ section (‘Data collection’ subsection) – P.9, lines 253-254. |
| **15** | Presence of non-participants | No. |
| **16** | Description of sample | Done.  Table 2 provides an overview of the demographics and practice profile of the study’s sample – See ‘Methods’ section (‘Participants and recruitment’ subsection) – P.10-11, lines 240-249. |
| ***Data collection*** | | |
| **17** | Interview guide | Done.  The semi-structured interview guide is presented in Additional file 2 – See ‘Methods’ section (‘Data collection’ subsection) – P.11, lines 254-258. |
| **18** | Repeat interviews | No. |
| **19** | Audio/visual recording | Done.  See ‘Methods’ section (‘Data collection’ subsection) – P.11, lines 262-264. |
| **20** | Field notes | Done.  See ‘Methods’ section (‘Data collection’ subsection) – P.11, lines 264-265. |
| **21** | Duration | Done.  **Recruitment duration:** See ‘Methods’ section (‘Participants and recruitment’ subsection) – P.9, lines 230-231.  **Data collection duration:** See ‘Methods’ section (‘Data collection’ subsection) – P.11, lines 252-253.  **Interview duration:** See ‘Methods’ section (‘Data collection’ subsection) – P.11, lines 253-254.  **Data analysis duration:** See ‘Methods’ section (‘Data analysis’ subsection) – P.12, lines 272-273. |
| **22** | Data saturation | No: In accordance with the Interpretive Description approach—which recognizes that subjective human experience can theoretically possess infinite variation—data saturation was not a desired outcome in this study. Rather, we focused on obtaining a deeper understanding of participants’ experiences, and on ensuring that the data we gathered was rich enough to answer our research question.  The decision-making process that occurred during data collection is fully described in the ‘Methods’ section (‘Participants and recruitment’ subsection) – P.10, lines 232-239. |
| **23** | Transcripts returned | No. |
| **Domain 3: Analysis and findings** | | |
| ***Data analysis*** | | |
| **24** | Number of data coders | One data coders (GC).  See ‘Methods’ section (‘Data analysis’ subsection) – P.12, lines 274-284. |
| **25** | Description of the coding tree | Done.  See ‘Methods’ section – P.12, lines 283-284 and Table 3 in the ‘Results’ section – P.14, starting from line 325. |
| **26** | Derivation of themes | Yes: An inductive approach to data coding was used. Themes were not identified in advance – See ‘Methods’ section (‘Data analysis’ subsection) –P.12, lines 270-272. |
| **27** | Software | Done.  See ‘Methods’ section (‘Data analysis’ subsection) – P.12, lines 275-278. |
| **28** | Participant checking | Yes: See ‘Methods’ section (‘Data analysis’ subsection) – P.12-13, lines 288-299. |
| ***Reporting*** | | |
| **29** | Quotations presented | Yes: Direct participant quotations were illustrated and identified (i.e., participant random number) in a table form – See Table 3 ‘Results’ section – P.14, line 330. |
| **30** | Data and findings consistent | Yes: Table 3 outlines excerpts from the interview transcripts (i.e., direct participant quotations) for each theme and sub-theme See ‘Results’ section –P.14, starting from line 325. |
| **31** | Clarity of major themes | Yes: In addition to Table 3, each theme (i.e., theme 1 to 4) is summarized in the second paragraph of ‘Results’ section (P.14, lines 322-328), and then further described in distinctive subsections of the ‘Results’ section:   - Theme 1 (P.14-16, lines 327-384); - Theme 2 (P.17-18, lines 385-427); - Theme 3 (P.18-19, lines 428-453); - Theme 4 (P.19-21, lines 454-500). |
| **32** | Clarity of minor themes | Yes:   - Relationships between themes and sub-themes are highlighted in Figure 1 – See ‘Results’ section – P.14, lines 319-321. - Excerpts from the interview transcripts (i.e., direct participant quotations) were provided for each sub-theme in Table 3 - See ‘Results’ section – P.14, lines 315-317; - In addition to Table 3, sub-themes are further described in distinct subsections of the ‘Results’ section: - Sub-themes 1.1 to 1.8 (P.14-16, lines 327-384); - Sub-themes 2.1 to 2.3 (P.18-19, lines 428-453); - Sub-themes 3.1 to 3.4 (P.18-19, lines 428-453); - Sub-themes 4.1 to 4.3 (P.19-21, lines 456-502). |

*Note*. CDs: Concurrent disorders; FRCPC: Fellow of the Royal College of Physicians of Canada; MD: Medical Doctor; MSc: Master’s in science; NA: Not applicable; No.: Item reference number; PhD: Doctor of Philosophy; QC: Quebec; RN: Registered nurse.
