# Supplementary material for: Experiences and perceptions of nurses participating in an interprofessional, videoconference-based educational programme on concurrent mental health and substance use disorders: a qualitative study
Source: BMC Nurs. 2022 Jul 4;21:177. doi: 10.1186/s12912-022-00943-w (PMC9251915; doi:10.1186/s12912-022-00943-w)
Supplement: Supplementary file 2 — Additional file 2. Individual semi-structured interview guidea [file 12912_2022_943_MOESM2_ESM.docx]

Additional file 2. Individual semi-structured interview guide^a^

| **Interview section** | **Prompts and guiding questions** |
| --- | --- |
| 1. Introduction | *In* *order to get to know you better, please start by telling me about your clinical practice as a nurse.*  Questions:   - In which healthcare setting do you currently work? - With which patient population do you currently work? - How would you describe your daily routine as a nurse (i.e., main activities, roles, and functions)? |
| 1. Learning objectives and experience in ECHO-CD | *Please explain what led you to sign up for the ECHO programme for CD management.*  Questions:   - What prompted or motivated you to sign up for ECHO-CD? - What is it about this programme that appealed to you? - What did you hope to get out of it? - What were you expecting as a participant?   *Please think about your overall participation in ECHO-CD.*  Questions:   - How would you describe your experience as a participant? - How would you describe your interactions with the experts and the other participants? - Have you presented a clinical situation during your participation? If yes, can you describe what took place during that virtual session? What has struck you the most about presenting a clinical situation? - Which aspects of the programme have you appreciated the most? - What have you less appreciated, or would you have changed about ECHO-CD? |
| 1. Perceived changes in competencies and clinical practice during ECHO-CD | *Please think about the skills and knowledge you think you may have acquired over your participation in ECHO.* Questions:   - What skills and knowledge do you believe you have acquired during your participation in ECHO-CD? - Which of these new skills and knowledge have you been able to apply into your clinical practice when attempting to care for individuals with CDs? - Can you provide me with examples of how you applied these new learning acquisitions into your clinical practice? |
| 1. Perceived factors that have facilitated or limited the development of competencies and their use in clinical practice | - In your opinion, what has been beneficial or helpful in supporting the development of your skills and knowledge in CD care? - What has been less beneficial? - What factors might have had an influence on your capacity to apply the skills and knowledge you believe that you have acquired during your participation? - Are there any circumstances or factors that affected your participation in ECHO-CD? |

*Note.* CDs: Concurrent disorders; ECHO-CD: Extension for Community Healthcare Outcomes programme for concurrent disorder management.

^a^The semi-structured interview guide was translated from French into English by a certified translator.
